# Tobacco Smoke Exposure and Lactation

**DOI:** 10.7759/cureus.73651

**Published:** 2024-11-13

**Authors:** Maria Vlachou, Giannoula A Kyrkou, Victoria Vivilaki, Vasiliki E Georgakopoulou, Paraskevi Katsaounou, Anastasia Κapetanaki, Athina Diamanti

**Affiliations:** 1 Department of Midwifery, Elena Venizelou, Athens, GRC; 2 Department of Midwifery, University of West Attica, Athens, GRC; 3 Department of Pathophysiology/Pulmonologist, Laiko General Hospital, Athens, GRC; 4 Pulmonary and Critical Care Department, Evaggelismos Hospital, National and Kapodistrian University of Athens, Athens, GRC; 5 Neonatology, Elena Venizelou Hospital, Athens, GRC

**Keywords:** breastfeeding support, lactation, maternity professionals, smoking cessation, tobacco smoke exposure

## Abstract

Tobacco smoke exposure remains a significant public health concern, particularly for lactating women and their infants. Despite widespread awareness of the harms of smoking during pregnancy, many women continue to smoke postpartum, directly impacting lactation success and infant health. Studies have shown that nicotine, the primary component of tobacco smoke, inhibits prolactin production and the milk ejection reflex, resulting in a decreased milk supply and poor breastfeeding outcomes. Additionally, the presence of harmful chemicals in tobacco smoke, such as cadmium and lead, can accumulate in breast milk, exposing infants to toxic substances with potential long-term health implications. Maternity professionals play a crucial role in supporting smoking cessation efforts among postpartum women, providing evidence-based counseling, resources, and referrals to cessation programs. This review aims to provide an update for maternity professionals on the effects of tobacco smoke exposure on lactation and breastfeeding outcomes. In this review, we will explore the physiological mechanisms through which tobacco smoke components can interfere with lactation. Furthermore, we will discuss the challenges faced by lactating women who smoke, including increased risk of mastitis, reduced breastfeeding duration, and impaired infant growth and development. Finally, we will highlight emerging research on novel interventions to reduce the adverse effects of tobacco smoke exposure on lactation, including pharmacological treatments and behavioral interventions tailored to postpartum women.

## Introduction and background

Breastfeeding improves both maternal and infant health while also promoting a strong bond between them. Infants who are exclusively breastfed for at least three months show reduced incidence of otitis media and respiratory infections and have fewer and less severe episodes of diarrhea [1]. Additionally, exclusive breastfeeding for at least four months is associated with a lower prevalence of allergies, asthma, and atopic dermatitis by the time the child is 2-3 years old, even for children with a family history of allergies [1]. Furthermore, exclusive breastfeeding continues to benefit children and adolescents by reducing the risk of obesity. In adulthood, it is associated with lower blood pressure and cholesterol levels [1]. Breastfeeding women also have a reduced risk of developing breast, ovarian, and endometrial cancers during the premenopausal period, as well as a lower risk of osteoporosis in the postmenopausal period [1]. Moreover, breastfeeding decreases maternal anxiety levels, fosters positive emotions, and strengthens maternal care for the infant [2,3]. Breastfeeding also helps regulate body weight, leading to postpartum weight loss and reducing the likelihood of conditions such as endometriosis, type II diabetes, osteoporosis, hypertension, cardiovascular diseases, metabolic syndrome, autoimmune diseases, multiple sclerosis, and Alzheimer's disease [2].

The World Health Organization (WHO) recommends exclusive breastfeeding for infants until six months of age, followed by age-appropriate complementary foods until two years of age or for as long as the mother-infant pair desires [4, 5]. A lack of education and knowledge about the art of breastfeeding can hinder its initiation, establishment, and continuation, often leading to premature cessation. For this reason, breastfeeding education during prenatal classes, along with support in the first hour after birth, during hospital stays, and through home visits throughout the postpartum period, can significantly increase the number of newborns who are exclusively breastfed in accordance with WHO recommendations [4,5].

Both primary and secondary exposure to cigarette smoke and the harmful substances it contains are responsible for the deaths of more than 8 million people globally each year [6]. Cigarette smoke contains over 4,000 chemicals toxic to the human body, of which more than 40 are known carcinogens [7]. Cigarette smoke contains some of these substances, while combustion produces others that trigger toxic reactions upon inhalation. Notable chemicals include nicotine, carbon monoxide, acetone, arsenic, benzene, cadmium, cyanide, formaldehyde, lead, mercury, nickel, phenol, and styrene [7]. Nicotine, a psychoactive and addictive component of tobacco, and carbon monoxide, when inhaled during the perinatal period through primary, secondary, or tertiary exposure, can cause alterations in the offspring. The placenta and breast milk also transfer these harmful compounds to the offspring [8].

Despite the well-documented research on the harmful effects of smoking on the health of offspring both prenatally and postnatally [9], many expectant and breastfeeding mothers continue to smoke during pregnancy and lactation.

The aim of this paper is to consolidate the scientific data on the effects of smoking during lactation and to serve as a learning resource and tool for providing quality care to midwives and obstetricians during the perinatal period, particularly in the lactation phase.

## Review

Detection of substances in breast milk: effects of nicotine and other substances on the components of breast milk

Smoking during lactation transfers substances found in tobacco, such as nicotine, to the breastfeeding infant. Nicotine levels peak within one hour of smoking and then gradually decrease [10]. Nicotine is considered an environmental pollutant capable of contaminating the atmosphere and soil [11]. The harmful effects of nicotine on breast milk depend on the number of cigarettes consumed by the breastfeeding mother and the time elapsed between the last cigarette and the start of breastfeeding [10,12].

The infant's digestive system absorbs nicotine from breast milk, which the liver then metabolizes into cotinine, a byproduct of nicotine metabolism [8]. Researchers have detected cotinine, the main metabolite of nicotine, in the urine of breastfed infants whose mothers smoke [10,13]. As the primary metabolite of nicotine, cotinine is used as a biomarker for tobacco smoke exposure (e.g., measuring cotinine levels in infant urine). Cotinine has a longer half-life (3-4 hours) in infants than in adults [8].

The detection of heavy metals (such as cadmium and lead) in colostrum and mature milk from women who smoke or are exposed to secondhand smoke demonstrates that tobacco use or exposure can alter the composition and nutrient content of human breast milk [14]. Milnerowicz & Chmarek [15] analyzed breast milk samples from smokers and non-smokers. Cadmium levels were found to be four times higher in the milk of smokers compared to non-smokers, while the concentration of total protein and metallothionein levels was lower in the breast milk of smokers. Thus, the researchers concluded that the breast milk of smoking mothers has a lower nutritional value compared to that of non-smoking mothers [15].

Changes in the composition and taste of breast milk due to smoking

Human breast milk is rich in essential components such as lactoferrin, oligosaccharides, polyunsaturated fatty acids, vitamins (vitamin C, vitamin E), minerals, immunoglobulins, enzymes, hormones, and growth factors. These components are crucial for supporting the healthy development of newborns. However, tobacco use and the harmful substances produced by smoking affect the quantity of these valuable components [8].

Researchers collected breast milk samples from breastfeeding mothers who smoke and those who do not and found that smokers' breast milk had lower concentrations of lipids and proteins, suggesting that smoking influences the alteration of breast milk composition [16-19]. Additionally, tobacco use inhibits hepatic lipoprotein lipase activity, impairs lipolysis, and increases maternal blood levels of cholesterol, triglycerides, and low-density lipoproteins [20]. Smokers' breast milk reduces the quantity of polyunsaturated fatty acids, especially omega-3 fatty acids [20]. Lipids are essential for the development of vision, brain, and body weight in infants [20]. The lower lipid content in the breast milk of smokers results in lower body weight, cognitive disorders, and limited physical growth in infants.

Another study notes that smoking reduces the iodine intake through breast milk [8]. Iodine deficiency results in the inability to synthesize thyroid hormones, which the infant's thyroid function depends on. It also causes sleep disturbances due to reduced sleep duration, intracellular oxidative damage, histopathological damage to the liver and lungs, reduced pancreatic beta cells, impaired glucose tolerance, increased body weight, brain damage, and reduced cognitive and motor function [8,21].

Colostrum and full-term milk from breastfeeding mothers have lower amounts of total equivalent antioxidant capacity (TEAC) and higher amounts of thiobarbituric acid-reactive substances (TBARS). They also have more antioxidant defense activity, which can be seen by higher levels of antioxidant enzyme activity [22,23]. As a result, smoking reduces antioxidant properties and alters immunological protections [18].

Finally, smoking and secondhand smoke exposure alter the taste of breast milk, making it difficult for the infant to breastfeed or leading to breastfeeding refusal [12,24]. It remains unknown whether similar taste changes occur in breast milk following cannabis use [25].

All of the above demonstrate that exposure to cigarette smoke in breastfeeding mothers affects the epithelial cells of the mammary gland, reducing milk production and altering its composition. This results in changes that diminish the protective properties of colostrum and mature milk, which serve as the primary nutrition for infants up to six months of age. These changes may have potential consequences for the health of the newborn and infant and reduce the beneficial effects provided by breastfeeding [8,26].

Effect of smoking on prolactin production

Studies demonstrate the risks associated with maternal smoking during lactation, which leads to a reduction in prolactin levels, decreased milk supply, and changes in both the composition and taste of the milk [12]. These effects often result in early weaning.

Factors related to the mother, the infant, and the environment influence the secretion of prolactin, the hormone responsible for producing breast milk during lactation. Prolactin deficiency can frequently cause lactation issues and disorders [27].

Cigarette smoke and nicotine alter prolactin levels in the bloodstream [18,28]. Consequently, they impair the stimulation of mammary gland development, reduce milk production, and inhibit lactation [24,28]. Additionally, prolactin levels decrease in non-smoking breastfeeding women exposed to secondhand smoke, leading to shorter or premature cessation of breastfeeding [24].

Impact of smoking on the process, duration, and effectiveness of breastfeeding

Among women who smoke, there is evidence of reduced milk production and shorter breastfeeding durations. Research shows that smoking mothers are less likely to initiate breastfeeding, and if they do, they breastfeed for shorter periods [29-31]. Furthermore, despite the intention to breastfeed, younger maternal age, lower educational levels, and smoking during pregnancy are associated with shorter breastfeeding durations [31,32].

Ariz et al. [32] monitored 399 pregnant women intending to breastfeed up to the 12th month postpartum. Results showed that participants who smoked during pregnancy frequently used pacifiers and bottle nipples and had lower rates of exclusive breastfeeding during the first week postpartum. Moreover, those who continued smoking during pregnancy introduced formula feeding earlier, specifically within the first month of life, compared to non-smokers who introduced formula at five months. The same study found that the average breastfeeding duration for smoking mothers was 90 days, while non-smokers breastfed for an average of 177 days, indicating that those who smoked had shorter breastfeeding periods. Additionally, the study noted that occasional smokers had longer breastfeeding durations compared to regular smokers [32]. Researchers concluded that smoking during pregnancy is associated with shorter breastfeeding durations, despite the mother's intention to breastfeed. They observed this association in the short term (up to six months), but not in the long term (up to 12 months when the study ended).

However, smoking cessation before or during pregnancy is associated with longer breastfeeding durations, suggesting that breastfeeding motivates smokers to modify their smoking habits and prevent relapse [32]. Even if they are unable to completely quit, many mothers reduce their smoking habits [33]. In a study by Joseph et al. [34], researchers aimed to assess factors related to breastfeeding intention, initiation, duration, and weaning among pregnant former smokers. The majority of participants quit smoking upon learning of their pregnancy and expressed a positive intention to breastfeed, with their desire to continue breastfeeding serving as motivation to remain smoke-free. However, some participants who relapsed and resumed smoking had shorter breastfeeding durations and weaned early [34].

Secondhand smoke exposure during pregnancy is associated with an increased likelihood of breastfeeding cessation and also affects breastfeeding duration in non-smoking mothers [35]. Additionally, postpartum women exposed to secondhand smoke are less likely to breastfeed [36]. A study by Rosen-Carole et al. [37] examined the effect of secondhand smoke exposure on breastfeeding duration and found that breastfeeding was shorter in these cases. Research links maternal exposure to secondhand smoke to an increased risk of exclusive breastfeeding cessation before six months, as well as reduced breastfeeding intention or cessation within the first two months of the newborn's life [38]. Smoking bans in the home and avoidance of secondhand smoke could potentially improve breastfeeding rates [39].

Researchers at Careggi University Hospital in Florence assessed the breastfeeding behavior, including nipple latch and swallowing, of 35 infants born to smoking mothers and 35 infants born to non-smoking mothers. After three months of follow-up, researchers concluded that infants of smoking mothers exhibited altered neurobehavioral profiles and had difficulty initiating and maintaining breastfeeding, with only 57.1% of infants of smokers successfully breastfeeding compared to 87.5% of infants of non-smokers [40].

These research findings are invaluable as they demonstrate the need for organized interventions aimed at smoking cessation, relapse prevention, increased breastfeeding initiation and duration, and promotion of exclusive breastfeeding, thus improving maternal and infant health [32,34]. Smoking mothers should be informed about the harmful effects of smoking, and the changes it causes in breast milk quantity and composition, and encouraged to change their smoking habits [41,42]. In cases where mothers choose to continue smoking while breastfeeding, they should receive specialized guidance from healthcare professionals to protect their infant's health and promote breastfeeding. Smoking mothers are advised to reduce smoking, ensure several hours pass between smoking and breastfeeding to minimize infant nicotine exposure, smoke in a separate room to protect the infant from secondhand smoke and wear a cover while smoking, which is removed afterward to protect the infant from thirdhand smoke exposure [42,43].

Healthcare providers must support and encourage breastfeeding even if the mother smokes, as the benefits of breastfeeding outweigh those of bottle-feeding and formula. Planning for relapse prevention early in the postpartum period and continuing it for up to three months after birth is ideal [44].

Effects of smoking on offspring

Epigenetics focuses on understanding the regulation of gene expression beyond what is encoded in DNA sequences and how environmental factors influence changes in gene expression. The placenta's epigenetic regulation mediates epigenetic alterations like miRNA imprinting and expression, as well as DNA methylation. Proper placental development and function are crucial for the growth and survival of the developing fetus. Investigating and understanding these epigenetic mechanisms is useful in identifying new biomarkers for exposure, burden, or disease risk [45].

The interaction between genes (during fetal development, the placenta) and the environment can lead to adverse conditions impacting both physical and mental health later in life [45]. The mother's use of cigarette smoke influences the expression of miRNA in the placenta during fetal development [46].

The fetal programming theory [47] identifies gene-environment interactions and explains how the intrauterine environment influences molecular aspects of development. Initially, researchers proposed a hypothesis linking the development of diseases in adulthood to an unfavorable nutritional environment for the fetus (poor maternal dietary choices during pregnancy). However, this risk may change if dietary habits improve postnatally. Beyond poor nutritional exposure, the adverse intrauterine environment is also linked to other health-threatening factors such as influenza, elevated stress levels, medications, maternal smoking, and secondhand smoke exposure [47].

Respiratory issues

Smoking during the perinatal period increases the incidence of respiratory diseases such as asthma, wheezing, bronchiolitis, and pulmonary disorders (e.g., impaired lung development or reduced lung function), leading to more frequent hospitalizations of children with smoking parents [9,48,49]. Maternal smoking and secondhand smoke exposure during pregnancy are associated with the development of asthma in childhood [50]. Vardavas et al. [51] found that children exposed to secondhand smoke during pregnancy were more likely to develop wheezing by age two compared to children not exposed to secondhand smoke. Postnatal exposure to secondhand smoke further increases the risk of wheezing in children.

Prematurity and physical development

Prenatal exposure to cigarette smoke (nicotine and carbon monoxide) hinders the exchange of nutrients and oxygen between the mother and fetus, restricting fetal growth. It has been found that smoking mothers give birth to newborns with low birth weights (with a reduction of 277 g compared to non-smokers or those who quit early in pregnancy) [9,29,38]. Mothers exposed to secondhand smoke during pregnancy are also more likely to give birth to low-birth-weight babies [52-54], smaller head circumferences [52], and premature infants [55].

Secondhand smoke exposure in infancy impacts growth. Baheiraei et al. [39] compared the weight, height, and head circumference of a sample of infants exposed to secondhand smoke with those who were not. Measurements were taken three times (5th-7th day of life, 2 months, and 4 months after birth). The researchers concluded that secondhand smoke exposure in infancy could lead to reduced weight and height during the first four months of life. Another study [56] investigated the interaction between smoking during pregnancy, preeclampsia, and birth weight. Results showed that women who developed preeclampsia and smoked during pregnancy had significantly smaller babies compared to those who did not develop preeclampsia and did not smoke.

Congenital anomalies and cleft palates

The occurrence of congenital anomalies in newborns has been associated with maternal smoking or secondhand smoke exposure during pregnancy [52]. Studies indicate that mothers who smoked or were exposed to secondhand smoke during pregnancy are more likely to have children with neural tube defects (e.g., anencephaly) or cleft lip with or without cleft palate [55,57-59].

Allergies

Secondhand smoke exposure may contribute to the development of childhood allergies due to disruptions in the immune system [60]. Damage to the epithelial cells of the nasal cavity, airways, and skin barrier can cause these disorders to affect the skin, such as atopic dermatitis, or the respiratory system, such as allergic rhinitis [60].

Obesity, metabolic syndrome, and overweight infants

Early and involuntary exposure of the fetus and newborn to cigarette smoke and nicotine (during pregnancy and breastfeeding) is linked to metabolic syndrome and obesity in childhood and adulthood [61]. The mechanism by which smoking during breastfeeding causes metabolic dysfunctions is not fully understood. Researchers concluded in a study of 378 adolescents prenatally exposed to maternal smoking that changes in brain synapses and reduced amygdala volume promote obesity [62].

Secondhand smoke exposure and non-exclusive breastfeeding are associated with infant obesity, while exclusive breastfeeding reduces the likelihood of obesity resulting from passive smoking [61]. Children exposed to smoke prenatally, whether through maternal smoking or secondhand exposure, exhibit a higher body mass index (BMI) in early childhood [63]. Teenagers whose mothers smoked or were exposed to secondhand smoke during pregnancy have a higher prevalence of obesity during adolescence [64]. The study found that not breastfeeding, maternal obesity, and excessive screen time contributed to adolescent obesity [64].

A study by Wen et al. [65] explored the relationship between cigarette smoke exposure through breast milk and weight gain risk. Wen et al. [65] used a sample of 21,063 mother-infant pairs, categorizing mothers by their smoking status (non-smokers, light smokers, moderate smokers, and heavy smokers) and recording the infants' feeding type (exclusive breastfeeding or bottle feeding) during daycare. After tracking the children's weight and height up to age seven and considering additional factors (maternal smoking, diet), the researchers concluded that cigarette smoke exposure through breast milk contributes to moderate weight gain by age seven, resulting in overweight children.

Sleep disorders

Sleep disturbances arise from nicotine's effects on brain function, its stimulating properties, and the inhibitory function of neurons that promote sleep, manifesting in irritability and crying [8]. Mennella et al. [12] observed that infants breastfed by mothers who had recently smoked had shorter sleep durations (53.4 minutes) compared to infants breastfed by mothers who abstained from smoking (84.5 minutes of sleep). The researchers concluded that the infant's sleep duration was shorter the higher the dose of nicotine the infant ingested through breastfeeding, depending on how close the mother had smoked to feeding.

Other problems

Additional problems for offspring resulting from maternal smoking and cigarette smoke exposure during the perinatal period include hearing loss [66], early childhood leukemia [67], cardiac rhythm disturbances [8,68], anemia, rickets [14], frequent colic episodes [8,9,69], sudden infant death syndrome (SIDS), excessive crying [70], paleness [8], hyperactivity and attention deficit disorder [71], memory deficits, and learning difficulties [8].

Cannabis and marijuana use during lactation

The legalization of cannabis (and marijuana) in many countries has led to the production of a wide variety of products for ingestion, topical application, inhalation, or vaping, with significantly higher potency than in the past [72]. Furthermore, the widespread belief in the safety of cannabis use has led to health issues in infants and children whose parents use it, as they are susceptible to its effects through breastfeeding and indirect exposure.

Research data indicate that the use of both tobacco and cannabis (either individually or together) during breastfeeding negatively impacts the developing brain of the newborn and infant [73]. It is associated with adverse pregnancy outcomes and health problems in infants and children, such as reduced muscle tone, decreased sucking reflexes [74,75], low body weight, reduced head circumference [66], increased likelihood of admission to neonatal intensive care units (NICUs) [76], delayed development [66], neurobiological development disorders, changes in normal brain function [66], sleep disorders, psychotic symptoms, learning difficulties, behavioral disorders (aggression, impulsivity), hyperactivity (ADHD), autism spectrum disorders [77], reduced cognitive ability, decreased gray matter [73,78], and increased risk of marijuana, alcohol, and opioid use during adolescence and young adulthood [73,79]. Furthermore, there are reports of negative academic performance [80]. The American Academy of Pediatrics and the American College of Obstetricians recommend avoiding the use of cannabis and cannabis-containing products throughout lactation due to potential neurodevelopmental issues in newborns and infants [81,82].

Researchers have detected delta-9-tetrahydrocannabinol (THC), a lipid-soluble psychoactive chemical in cannabis, in breast milk, with concentrations peaking one hour after cannabis inhalation and gradually decreasing over the next four hours [83]. Mothers who smoked cannabis detected THC in breast milk at concentrations 7.5 times higher than in their plasma, and these mothers also detected it in the feces and urine of their breastfed infants [84]. Other studies [85,86] have similarly confirmed THC in breast milk, thus showing its transfer to breastfeeding infants.

Further research is necessary to assess the safety of cannabis use during pregnancy and breastfeeding in order to reduce unintended health consequences for infants and children from cannabis use. This research should investigate its impact on neurodevelopment, sleep, feeding, and cognitive development beyond the first year of life [73-75]. Avoid using cannabis as a treatment for hyperemesis gravidarum (severe morning sickness) [87].

Healthcare professionals hold varying opinions regarding the breastfeeding practices of mothers who use cannabis. Scientific guidelines recommend avoiding cannabis use during lactation. Additionally, mothers who use cannabis should receive full disclosure about the negative effects (such as developmental disorders and increased risk of infant death) of exposing a breastfeeding infant to cannabis through breast milk and secondhand exposure, including from a father or other household members [81,88-90].

There is currently a lack of scientific and research data on the transfer of cannabis to amniotic fluid and human breast milk [87,91], and we do not yet know the safe levels of use during the perinatal period [87]. Healthcare professionals should equip themselves with the necessary knowledge to provide expectant parents and breastfeeding women with appropriate, evidence-based recommendations about the risks of intrauterine exposure and potential harm to the developing brain. These professionals should also encourage pregnant and breastfeeding women to avoid cannabis use throughout the perinatal and lactation periods or reduce usage if complete cessation is not feasible [91]. To minimize cannabis exposure through breast milk, breastfeeding should be avoided within one hour of cannabis use [91], and efforts should be made to prevent infants and children from being exposed to such substances [87,92-94].

New Tobacco Products

Vaping Devices: E-Cigarettes and ENDS

E-cigarettes and other electronic nicotine delivery systems (ENDS) are battery-operated devices that heat a liquid (usually glycerin or propylene glycol), creating vapor. The liquid often contains nicotine, flavorings (fruity or sweet), additives, and various pollutants and carcinogenic substances [42,95]. The tobacco residues from e-cigarettes can be deposited on surfaces in the environment, creating thirdhand exposure risks for offspring with potentially harmful health consequences [96]. Similar risks arise from secondhand exposure to e-cigarette vapor, which children can inhale, ingest, or absorb through the skin long after the device's use [96].

Despite these risks, people frequently use these devices as smoking cessation tools to switch from conventional cigarettes [97]. Additionally, breastfeeding smokers may believe that e-cigarettes are safer during lactation compared to conventional cigarettes, which leads them to continue breastfeeding while using them [98].

Heated Tobacco Products

Currently, no research has examined the relationship between heated tobacco use and breastfeeding. However, concerns about breastfeeding as a smoker, especially regarding the potential contamination of breast milk with heated tobacco, and related chemicals-are not entirely unwarranted. Although smoking mothers are advised that it is safer to breastfeed than to use formula, exposure to harmful toxins remains a risk, as these may transfer to infants through breast milk. This transfer occurs due to the specific mechanisms involved in breast milk production and secretion. While heated tobacco products contain significantly fewer toxicants than traditional cigarettes, assessing how these substances might transfer to breast milk compared to traditional cigarettes could provide women with the clear, straightforward information they seek [99]. 

Smokeless Tobacco (SLT)

The term "smokeless tobacco" (SLT) refers to tobacco products that do not produce smoke when smoked or burned [99]. It is used in various forms, such as chewing, nasal inhalation, or placement between the gums, cheeks, or lips [99]. SLT contains nicotine and other harmful and carcinogenic chemicals, leading to addiction [100].

Researchers observed that different life stages, including adolescence, pre- and post-marriage periods, as well as familial and social environments, influence the use of SLT in a study by Singh et al. [101], which involved 20 pregnant and 22 breastfeeding women. The study also revealed that participants were aware of the harmful effects of SLT during pregnancy but were unaware of its consequences during breastfeeding. Many of the participants expressed a desire to quit smoking but noted a lack of knowledge on how to do so. These findings highlight the need for smoking cessation intervention tools specifically tailored for groups such as pregnant and breastfeeding women.

Snus (Nicotine Pouches)

Snus is a smokeless tobacco product consumed orally by placing it behind the lips. It contains nicotine, causes addiction, and has harmful health effects. Its use during pregnancy and lactation can result in adverse effects on the developing fetus and newborn, such as premature birth, fetal and neonatal mortality, and small-for-gestational-age infants [43]. The use of snus before and during pregnancy increases the likelihood of continued use during breastfeeding [102].

Researchers discovered through ultrasound that children exposed to snus in utero, due to maternal use, had increased carotid artery thickness and stiffness during preschool years in the study by Nordenstam et al. [102]. Due to the nicotine content, snus can disrupt prolactin secretion, leading to breastfeeding duration issues and affecting the quantity and composition of breast milk [8]. However, the study by Kreyberg et al. [43] found no significant results regarding the impact of snus use on breastfeeding.

Waterpipe (Hookah)

A study analyzing data from two national health surveys in Jordan involved a total of 6,726 mother-infant pairs (infants under 25 months) [103]. Among infants aged 0-6 months, 87% were breastfed, compared to 19.4% of infants aged 18-24 months. Of the mothers who participated in the study, 4.4% smoked conventional cigarettes, 5.4% smoked hookah, and 1.6% smoked both. In Jordan, people commonly use hookah, either in conjunction with conventional cigarettes or on its own. The researchers found that 57.7% of breastfed infants had non-smoking mothers, while wives who smoked conventional cigarettes, hookah, or both breastfed at lower rates [103]. Therefore, smoking was associated with lower breastfeeding rates up to two years of age [103].

Nicotine replacement therapy (NRT) and pharmacotherapy

NRT, a non-pharmacological method, aids in the cessation of smoking. There are short-acting and long-acting formulations available, and smokers can access them in the form of patches, gum, oral sprays, or inhalers [42]. However, research data regarding the use of NRT during breastfeeding is limited. In the study by Kreyberg et al. [43], the researchers did not find significant results regarding the use of nicotine substitutes during breastfeeding.

Nicotine and cotinine, the primary metabolites of nicotine, are detectable in breast milk. Additionally, cigarette smoke contains a variety of potentially harmful substances that can pass through breast milk, causing harm to infants [8]. Given this, using nicotine substitutes can shield infants from the harmful substances present in cigarette smoke [104].

Nicotine absorption from NRT appears to be at lower concentrations in the circulation of breastfeeding smokers compared to nicotine from smoking, and these concentrations are likely proportionally lower in breast milk [42]. However, this does not hold true for nicotine patches, as they seem to increase the levels of nicotine in the blood of breastfeeding women to a level comparable to smoking [42].

For smoking cessation, doctors also prescribe bupropion, an antidepressant, and varenicline, another pharmaceutical agent. Breastfeeding contraindicates the use of both medications [33].

The role of healthcare professionals

Healthcare professionals should advise breastfeeding mothers to quit smoking and protect themselves from secondhand and thirdhand smoke, given the potential risks of cigarette smoke exposure to infants and the changes in breast milk composition caused by smoking. In cases where breastfeeding smokers are unable to quit, healthcare professionals should provide guidance on reducing or adjusting smoking habits during breastfeeding.

Healthcare professionals who specialize in perinatal care play a critical role in protecting pregnant women, postpartum mothers, and their infants from smoking-related harm [105]. Understanding healthcare professionals' experiences with providing smoking cessation support during the perinatal period can help inform intervention design and improve routine care [106].

Midwives, in particular, are the primary healthcare providers responsible for caring for the mother-infant dyad throughout the perinatal period. Through frequent contact with midwives, women have opportunities to receive information about the harms of smoking for both their health and their baby’s health. Midwives can support them in their efforts to quit smoking without compromising their overall perinatal care [105]. Midwives are also essential in promoting maternal and child health through actions that support breastfeeding, smoking cessation, and relapse prevention [38].

Smoking cessation interventions provided by midwives in the context of perinatal care can help women reduce their smoking initially and eventually quit altogether while also facilitating breastfeeding initiation and continuation.

The research team of Naughton et al. [107] aimed to evaluate the interest, use, and attitudes toward smoking cessation support during pregnancy and postpartum among women who smoke or had recently quit. Their results indicated that interest in attending a smoking cessation intervention program was high during the later stages of pregnancy and immediately after birth but significantly decreased by three months postpartum. Additionally, fewer than half of the women who smoked reported speaking with a midwife about smoking cessation early in pregnancy, revealing a lack of healthcare professional training in this area [107].

In the study by Philips et al. [108], semi-structured phone interviews were conducted to identify barriers reported by pregnant and postpartum smokers and former smokers that made it difficult for them to quit smoking or led to relapse. Among the barriers mentioned was the lack of support from healthcare professionals.

It was also evident that although healthcare professionals working in the perinatal period typically ask about tobacco use during initial history taking, they do not provide systematic counseling for smoking cessation throughout the perinatal period. As a result, women who want to change their smoking habits may not find adequate support [109].

Some women expressed concerns and were reluctant to use nicotine replacement therapy for relapse prevention, fearing that their nicotine addiction would persist [108]. However, they appeared more open to the idea if a healthcare professional recommended it.

Thus, smoking cessation and relapse prevention support from healthcare professionals should continue during the postpartum period, extending to the entire family [108,110]. Smoking cessation programs tailored specifically for the perinatal period should be implemented to ensure relapse prevention and the identification of motivating factors [111].

From the perspective of healthcare professionals, barriers to smoking cessation support include the need to protect the professional-client relationship, lack of knowledge and confidence regarding smoking cessation interventions during the perinatal period, unfamiliarity with tools (such as the Fagerstrom test), time constraints, service priorities, limited or nonexistent funding for intervention programs, resistance from women to quitting smoking, and difficulty in approaching the topic [112-115].

Despite these challenges, healthcare professionals generally encourage women to quit smoking early in pregnancy during their initial history-taking, emphasizing the potential harm to both mother and baby. However, professionals often feel they lack the necessary training and knowledge to provide ongoing support or interventions during pregnancy or postpartum [107,112,113,115,116].

A study of 296 pediatricians revealed that while over half felt confident in advising mothers on breastfeeding, they lacked the knowledge to counsel breastfeeding smokers, underscoring the need for pediatricians to receive training in topics related to lactation, tobacco use, and smoking cessation programs [117].

## Conclusions

Researchers have detected nicotine in the breast milk of smoking mothers during lactation. This finding indicates that there may be critical health impacts on the newborn and infant, as breast milk serves as the primary source of nutrition for the first six months of life. Furthermore, not only can breast milk expose an infant to cigarette smoke compounds, but also secondhand and thirdhand smoke, both of which carry negative health implications.

The majority of research findings underscore the need for policies that implement specific educational programs aimed at minimizing smoke exposure and promoting smoking cessation during pregnancy and breastfeeding. These programs should address cessation motivations, the challenges posed by the limited availability of pharmacotherapy during lactation, and the impacts of smoking on breastfeeding.
